# Supplementary material for: Development and validation of a self-administered computerized cognitive assessment based on automatic speech recognition
Source: PLoS One. 2024 Dec 16;19(12):e0315745. doi: 10.1371/journal.pone.0315745 (PMC11649080; doi:10.1371/journal.pone.0315745)
Supplement: Attachment — Submitted filename: Response to Reviewers #2.docx [file pone.0315745.s001.docx]

**Detailed Responses to Reviewer #2**

Dear Reviewer,

We really appreciate the reviewer’s detailed comments on our manuscript. The reviews have raised some excellent questions, and we sought to address each issue in detail in this letter and in the attached revised manuscript. You will find the reviewer’s comments in *italic*, followed by our detailed response. The original text is shown in blue, and the revised or added text is shown in red.

We hope that you will find our responses to be adequate. We thank you again for your time and effort spent reading and commenting on our manuscript.

Kind Regards,

Min Woo Oh

Jeonghwan Lee

November 22, 2024

1) *Data availability statement need to clearly mentioned.*

: Thank you for your comment regarding the data availability statement. We acknowledge the importance of clearly specifying how the research data can be accessed.

Initially, we stated in the manuscript that "All relevant data are within the manuscript and its Supporting Information files." However, after further discussion with the Institutional Review Board (IRB) overseeing this study, we determined that the dataset contains potentially sensitive information about participants. To protect participant confidentiality, it was decided that data sharing should be facilitated through the IRB for researchers who meet the criteria for accessing confidential data.

Researchers who wish to access the data may contact the Chungbuk National University Hospital Ethics Committee via telephone at +82-43-269-6771 to request permission and further details regarding the access procedures. This revised approach has been updated in the "Data Availability" section of the online submission process under "Additional Information."

*2) It was a bit confusing that that is an automated system or needs human labelling, make it clear.*

: Thank you for your comment regarding the clarity of whether CogMo operates as a fully automated system or requires human labelling. We appreciate the opportunity to provide further clarification.

CogMo is designed to function as a fully automated system. Participants' verbal responses are transcribed into text using the Google Cloud's Speech-to-Text application programming interface. These transcriptions are then processed through the CogMo scoring framework, which automatically evaluates the responses based on predefined criteria. At no point in this process is human labelling or manual intervention required for scoring.

To ensure the accuracy of the automated system in this study, we validated the ASR-generated transcriptions by comparing them with manually transcribed data. The manual transcriptions were created by examiners who listened to the recorded audio responses provided by participants during the assessment, ensuring the reliability of the scoring process. However, this manual validation was conducted solely to confirm the accuracy of CogMo's ASR system and was not part of its routine procedures.

We have updated the manuscript to clearly state that CogMo operates as a fully automated system for data processing and scoring.

[Line 124-126]

The participants performed the CCT by touching the screen of tablet PC or verbally answering the test questions, and their responses were scored automatically.

🡪

The participants performed the CCT by touching the screen of the tablet PC or verbally answering the test questions. Verbal responses were transcribed using the Google Cloud’s speech-to-text API and automatically scored through the CogMo scoring framework, which evaluated the accuracy and relevance of the responses based on predefined criteria.

*3) How the scoring of different component will be done automatically in the future, through analysis of facial and spoken content or eye tracking devices.*

: Thank you for your insightful comment regarding the potential for further automating the scoring of different components in the future. We appreciate the opportunity to elaborate on how technologies such as facial analysis, spoken content, and eye tracking devices might enhance CogMo’s capabilities.

Currently, CogMo is designed to score cognitive performance based on participants’ verbal responses using an automatic speech recognition (ASR) system integrated with a scoring framework. However, we acknowledge the potential for integrating additional modalities in the future to further enhance its diagnostic accuracy and comprehensiveness.

As part of another ongoing study, we are currently developing an app to evaluate post-stroke language disorders, such as aphasia and dysarthria, using a computerized approach instead of traditional assessments conducted by speech therapists. This development involves applying motion analysis to image data, including the analysis of lip movements and facial expressions. These technologies align with the reviewer’s suggestion and could be applied in the future not only to ASR but also to the analysis of various components, enhancing the effectiveness of cognitive impairment diagnosis.

We believe that integrating such advanced technologies into CogMo holds great promise for creating a more comprehensive and multidimensional cognitive assessment tool. Future studies will explore these possibilities to further enhance CogMo’s capabilities.

*4) making it fully automatic will lead to reduction in results, please discuss.*

: Thank you for your insightful comment regarding the differences between manual and automatic scoring observed in our results, as presented in Table 3. We appreciate the opportunity to address this important aspect of our study.

The differences in scores between manual and automatic scoring can be attributed to the inherent characteristics of the automated system. The automated scoring system strictly adheres to predefined rules, evaluating responses solely based on these criteria without the flexibility of human judgment to account for partial responses or ambiguous cases that might receive partial credit in manual scoring. Additionally, automatic transcription errors, particularly for unclear speech or dialectical variations, may contribute to reduced scores in the automatic scoring process. These limitations are inherent to ASR-based systems and likely explain the overall lower scores observed in the automated results.

Despite these differences, the relative trends between groups (normal vs. CI) remained consistent across both scoring methods. This indicates that while the absolute scores differed, the automated system effectively preserved the ability to distinguish between the two groups. Furthermore, the observed reduction in scores with automatic scoring does not undermine the system's diagnostic utility but highlights the importance of refining the ASR system to further minimize such discrepancies.

We have discussed these findings in the revised manuscript's discussion section to provide greater clarity and context. Additionally, we plan to explore ways to improve the accuracy of the automatic scoring system in future studies, including enhanced ASR algorithms.

[Line 211-215]

CogMo features better diagnostic accuracy than existing CCTs because it supports an input interface with voice recognition, unlike existing CCTs that employ touch-based input interfaces, such as touch screens, mouse/keyboard [27]. Therefore, it can be used to evaluate cognitive function in a manner similar to face-to-face tests, such as MMSE and MoCA, which are administered in clinical practice.

🡪

CogMo demonstrates enhanced diagnostic accuracy compared to existing CCTs by incorporating a voice recognition input interface, whereas most existing CCTs rely solely on touch-based input methods, such as touch screens or mouse/keyboard [38]. This feature allows CogMo to assess cognitive functions in a manner that closely resembles face-to-face tests commonly used in clinical practice, such as the MMSE and MoCA, while maintaining the advantages of a computerized approach. However, the automated scoring system strictly adheres to predefined rules, which may result in slightly stricter scoring compared to manual scoring. This difference can be attributed to the inherent limitations of ASR systems, such as transcription errors caused by unclear speech or dialectical variations. Despite these differences, the relative trends between the two groups based on CI remained consistent, supporting the diagnostic utility of CogMo.

5) *MMSE prediction is also performed through speech of picture description tasks, please discuss and add references.*

: Thank you for your comment regarding the use of picture description tasks for MMSE prediction and the need for additional discussion and references.

Studies such as Hernández-Domínguez et al. (2018) and Kavé & Dassa (2018) have demonstrated the utility of picture description tasks in identifying cognitive impairments, particularly in individuals with Alzheimer’s disease (AD) and mild cognitive impairment (MCI). For example, Hernández-Domínguez et al. (2018) utilized automated systems to evaluate linguistic and phonetic features during the Cookie Theft picture description task, identifying significant correlations between these features and MMSE scores. Similarly, Kavé & Dassa (2018) analyzed lexical and grammatical aspects of picture descriptions, finding strong associations between MMSE scores and specific language impairments, such as reduced lexical diversity and information content.

These studies highlight the potential of picture description tasks as effective tools for assessing cognitive function, with automated systems enhancing objectivity and scalability. While these methodologies provide valuable insights, our study takes a different approach. CogMo employs structured, language-based tasks specifically designed to evaluate multiple cognitive domains in a standardized manner using automatic speech recognition (ASR) technology. Unlike open-ended picture description tasks, which often rely on episodic memory and spontaneous speech, CogMo focuses on tasks that minimize variability across participants and emphasize accessibility for diverse populations. This structured approach ensures consistency and scalability while maintaining diagnostic utility.

CogMo’s ASR technology could also be adapted to support open-ended language function tests, such as picture description tasks. These tasks are well-established for their strong association with cognitive impairment, particularly within the language domain. Integrating such assessments into CogMo has the potential to further enhance its diagnostic accuracy by capturing richer linguistic data and providing a more comprehensive evaluation of cognitive functions.

We have incorporated these references and discussions into the revised manuscript to provide greater context and clarity regarding the differences and potential advancements in cognitive assessment methodologies.

[Line 250-252]

Third, as some individuals may be reluctant to have their cognitive functions assessed in a formal clinical setting, a computerized test may make them more receptive to subsequent formal clinical assessments [39].

🡪

Third, as some individuals may be reluctant to have their cognitive functions assessed in a formal clinical setting, a computerized test may make them more receptive to subsequent formal clinical assessments [50]. Lastly, CogMo’s ASR technology could be adapted to incorporate open-ended tasks, such as picture description, which previous studies have shown to be closely associated with CI, particularly in the language domain [51, 52]. Such adaptations could enable the evaluation of richer linguistic data and a broader range of cognitive functions, potentially enhancing its diagnostic accuracy.

6) *Introduction is lacking discussion about the prediction of MMSE in other languages.*

: Thank you for your insightful comment regarding the discussion of MMSE prediction in other languages. We acknowledge the importance of addressing how the MMSE has been validated and utilized across different linguistic and cultural contexts.

In response to your comment, we have revised the Introduction to include a discussion highlighting the MMSE's adaptability and diagnostic utility in various languages. Specifically, we now state that the MMSE has been translated and adapted worldwide, with multiple validation studies consistently demonstrating its high diagnostic value for detecting cognitive impairment. These studies underscore the robustness of the MMSE as a screening tool, regardless of language differences, supporting its applicability across diverse populations.

This addition provides a more comprehensive background for our study, which uses the MMSE as a reference standard for validating CogMo. By referencing the MMSE's strong diagnostic performance in different linguistic settings, we aim to highlight its relevance and applicability as a gold standard for cognitive screening.

We believe these revisions address your comment and provide greater context regarding the MMSE's role in predicting cognitive impairment across languages.

[Line 81-82]

Therefore, simple cognitive screening assessment tools such as the mini–mental state examination (MMSE) and Montreal cognitive assessment (MoCA) are widely used to screen for CI [14].

🡪

Therefore, simple cognitive screening assessment tools, such as the mini–mental state examination (MMSE) and Montreal cognitive assessment (MoCA), are widely used to screen for CI [14]. In particular, the MMSE has been translated and adapted into various languages across different countries, with multiple validation studies demonstrating its high diagnostic value for detecting CI, regardless of language differences [15].

7) *The limitation of study is Korean language, please discuss.*

: Thank you for your insightful comment regarding the language-specific limitation of our study. We recognize that conducting the study in Korean poses certain constraints, particularly concerning the generalizability of our findings to populations speaking other languages.

CogMo was designed and validated in Korean to ensure that the test items and instructions were culturally and linguistically appropriate for the target population. However, we acknowledge that linguistic and cultural differences can influence cognitive assessments. For example, language-based tasks, such as verbal fluency or sentence repetition, may yield different outcomes depending on the structure and characteristics of the language used. These factors must be carefully considered when applying CogMo in other linguistic and cultural contexts.

That said, some of CogMo’s subtests, such as "finding a puppy," "matching the shadows," "ordering the light," and "counting numbers," which are designed to evaluate cognitive domains beyond language, are less dependent on the Korean language. With minor adaptations, these subtests could potentially be applied to individuals speaking other languages, making CogMo more versatile across diverse populations. This flexibility is one of the key strengths of CogMo.

To address this limitation, further studies should aim to adapt and validate CogMo for use in other languages and cultural settings. This process would involve translating and culturally adapting the language-based test items, followed by validation studies to ensure the tool's diagnostic accuracy and reliability across diverse populations. By doing so, we hope to extend the utility of CogMo beyond Korean-speaking populations and enhance its applicability as a universal cognitive assessment tool.

[Line 262-263]

Finally, some cognitive functions (orientation and calculation) could not be evaluated because CogMo does not have test items related to these functions.

🡪

Finally, CogMo was developed and validated in Korean, which presents a limitation in generalizing the findings of this study to other linguistic and cultural contexts. However, certain subtests, such as finding a puppy, matching the shadows, and counting numbers, assess cognitive domains beyond language and are less dependent on the Korean language. With minor adaptations, these subtests could be applied to other linguistic groups, expanding CogMo’s applicability across diverse populations.

8) *What speech recognition models are used (Whisper is teh state of the art but requires resources), and how it affects the deployment of those models in low-resource dives like tablets or mobile phones. Please discuss.*

: Thank you for your insightful comment regarding the speech recognition models used in our study and their applicability in low-resource environments like tablets or mobile phones. We appreciate the opportunity to clarify and expand on this important aspect.

In this study, we utilized Google Cloud’s Speech-to-Text API for speech recognition, a widely used cloud-based ASR solution. This API is powered by deep learning models that offer robust transcription accuracy across various acoustic environments and speaker characteristics. While models like Whisper represent state-of-the-art ASR technology with exceptional performance, they often require substantial computational resources for local deployment, making them less practical for real-time applications on low-resource devices like tablets or mobile phones.

By contrast, Google Cloud’s Speech-to-Text API utilizes cloud computing, which reduces the computational burden on local devices. This approach allows CogMo to operate efficiently on low-resource platforms by offloading the heavy processing tasks to the cloud. However, this reliance on cloud infrastructure may present limitations in environments with poor internet connectivity or restricted access to cloud services.

In terms of deployment on low-resource devices, future iterations of CogMo could explore alternative approaches, such as fine-tuning lightweight on-device ASR models optimized for specific tasks or integrating hybrid systems that combine on-device preprocessing with cloud-based transcription. In parallel, we are developing a computerized aphasia and dysarthria assessment application for patients with post-stroke language disorders, which incorporates a custom-developed on-device ASR model. We plan to adapt this model for future use in CogMo, further enhancing its functionality and applicability across diverse environments.

9) *Also in introduction discussion, what advantages CogMO is brining for elders, is there any evidence to prove it is better than MMSE.*

: Thank you for your insightful comment regarding the advantages of CogMo for older adults and the need to provide evidence of its superiority over the MMSE. We appreciate the opportunity to address these important aspects in the Introduction section.

CogMo was specifically designed to address limitations commonly associated with traditional paper-based cognitive assessments like the MMSE, particularly when administered to older adults. One significant advantage of CogMo is its use of pre-recorded visual and auditory instructions, which ensure consistent guidance during the testing process. This feature minimizes variability caused by testers' instructional bias and reduces anxiety among older adults who may be unfamiliar with traditional testing environments. Additionally, CogMo’s automated scoring system eliminates human error, enhancing reliability and standardization.

Another advantage is CogMo’s voice interface, which allows individuals, including those with limited literacy or unfamiliarity with digital devices, to independently complete the test. This is particularly beneficial for older adults, as it reduces their reliance on external testers and creates a more accessible and user-friendly testing experience.

While the MMSE has been widely validated and remains a gold standard for cognitive screening, it primarily relies on manual administration and scoring, which can introduce variability. In contrast, CogMo utilizes advanced technologies, such as automatic speech recognition (ASR), to provide a more streamlined and objective assessment process.

Evidence supporting these advantages is drawn from the current study. For instance, CogMo demonstrated high reliability, with strong correlations between manual and automatic scoring (r = 0.98). Moreover, the automated nature of CogMo allows for multi-domain cognitive assessments, including domains like language and execution, which are not comprehensively covered by the MMSE. This expanded scope offers a more holistic evaluation of cognitive function, which is particularly important for identifying non-memory-related cognitive impairments among older adults.

These aspects highlight CogMo’s unique advantages for older adults and its potential as a superior alternative to traditional cognitive screening tools like the MMSE. We have incorporated these points into the revised manuscript to provide greater clarity and context.

[Line 92-94]

Therefore, we developed CogMo, a CCT that assesses language-related cognitive functions using a voice interface with automatic speech recognition (ASR), organizes test content into familiar topics for older adults, and allows illiterate individuals to take the test independently.

🡪

Therefore, we developed CogMo, a CCT designed to overcome the limitations of traditional cognitive assessments. CogMo evaluates language-related cognitive functions using a voice interface with ASR, ensuring accessibility for older adults, including those with limited literacy. It also organizes test content into familiar topics to enhance engagement, making the test more user-friendly for older populations.
